# Increasing Completion Rate and Benefits of Checklists: Prospective Evaluation of Surgical Safety Checklists With Smart Glasses

**DOI:** 10.2196/13447

**Published:** 2019-04-29

**Authors:** Thomas Boillat, Peter Grantcharov, Homero Rivas

**Affiliations:** 1 School of Medicine Stanford University Stanford, CA United States; 2 Data Science Institute Columbia University New York, NY United States; 3 Design Lab College of Medicine Mohammed Bin Rashid University of Medicine and Health Sciences Dubai United Arab Emirates

**Keywords:** smart glasses, surgical safety checklists, surgery, usability, time-out event

## Abstract

**Background:**

Studies have demonstrated that surgical safety checklists (SSCs) can significantly reduce surgical complications and mortality rates. Such lists rely on traditional posters or paper, and their contents are generic regarding the type of surgery being performed. SSC completion rates and uniformity of content have been reported as modest and widely variable.

**Objective:**

This study aimed to investigate the feasibility and potential of using smart glasses in the operating room to increase the benefits of SSCs by improving usability through contextualized content and, ideally, resulting in improved completion rates.

**Methods:**

We prospectively evaluated and compared 80 preoperative time-out events with SSCs at a major academic medical center between June 2016 and February 2017. Participants were assigned to either a conventional checklist approach (poster, memory, or both) or a smart glasses app running on Google Glass.

**Results:**

Four different surgeons conducted 41 checklists using conventional methods (ie, memory or poster) and 39 using the smart glasses app. The average checklist completion rate using conventional methods was 76%. Smart glasses allowed a completion rate of up to 100% with a decrease in average checklist duration of 18%.

**Conclusions:**

Compared with alternatives such as posters, paper, and memory, smart glasses checklists are easier to use and follow. The glasses allowed surgeons to use contextualized time-out checklists, which increased the completion rate to 100% and reduced the checklist execution time and time required to prepare the equipment during surgical cases.

## Introduction

### Background

Reducing complications and deaths in operating rooms (ORs) due to human error is a big challenge for hospitals. To explain variability in surgical outcomes, studies have primarily focused on patient pathophysiological risk factors and surgeon skills [1]. Hence, when the patient did not account for the surgical complication, the error was then attributed to the surgeon’s aptitudes and capabilities [2]. However, more recent research shows that errors “arise not from the solitary actions of individuals but from conflicting, incomplete, or suboptimal systems [3].” These systems notably refer to the people involved in surgical cases as well as the tasks, tools, or technologies; environment; and organization (eg, hospitals or clinics).

To analyze and improve patient safety, the US Institute of Medicine and the National Academy of Engineering have promoted the use of human factor techniques. Human factor techniques investigate factors and develop tools that facilitate the achievement of goals (eg, reduce errors, increase productivity, improve safety) [4]. In this view, among many different initiatives, the World Health Organization (WHO) has collected scientific evidence and published guidelines to address part of the problems with safety of surgical patients [5]. These guidelines are summarized as a 19-item checklist that aims to establish systematic verifications before anesthesia, before surgery, and after surgery [6]. Existing research demonstrates that when systematically applied, surgical safety checklists (SSCs) can reduce complications and mortality from 19.9% to 11.5% and 1.6% to 1.0%, respectively [7,8].

However, at the expense of patient safety, SSCs have not been entirely adopted by hospitals. In the United Kingdom and France, where the use of the SSC is mandatory, the average SSC completion rate is 60% [9,10]. Such low completion rates are notably explained by the OR’s constraints and bad design of the SSC implementations (eg, the checklists are often formatted by administrative staff who do not have the required skills; as a result, checklists are often difficult to read due to inappropriate fonts and colors) [4].

This is particularly the case with the time-out checklist. Because it takes place right before surgery commences and surgeons are not supposed to leave the 30 cm (12-inch) sterile area around the surgical table [11], they have to rely on a poster on the OR wall, often far from their field of view [9]. Additionally, surgeons complain that time-out checklists are not specific enough and often require them to spend time verifying irrelevant things. Finally, checklists are sometimes obsolete, and checklist completion is often not documented in the patient’s medical record. Consequently, time-out checklists often do not bring enough benefits to surgeons and, thus, are not systematically used [12,13].

In other fields where checklists are heavily used, information technologies such as mobile devices are very often solicited to improve checklist execution [14]. Existing literature shows that information technology can enhance checklist support by reducing human error and increasing safety [15]. Given that surgeons must not touch nonsterile equipment, the use of mobile devices in not optimal. However, smart glasses, which have recently become available on the market, present an interesting alternative, and surgeons have already begun to investigate their potential. While recent research has demonstrated the benefits of using smart glasses in ORs [16-18], no study has empirically investigated their use to execute checklists.

With this study, we investigate the following: *Are smart glasses a potential technology to use to execute SSCs?* Given that surgeons complain about the rigidity of traditional checklists, we also aim to evaluate the following: *How can smart glasses bring more benefit to SSCs?* To answer these two questions, the authors designed and evaluated a checklist app for smart glasses that was implemented over 6 months. Our results demonstrate that time-out checklists executed on smart glasses are easy to read, follow, and execute. When contextualized for a specific surgery, smart glasses can increase the time-out checklist completion rate to 100% while saving time in execution and preparation.

### Existing Research: Smart Glasses as Candidates for Use During Surgical Safety Checklists

Smart glasses are a wearable technology that uses spectacle frames to display contextualized information in a person’s field of view [19]. The main piece of hardware is a head-mounted display that allows the user to access texts, pictures, and videos. The glasses are also equipped with a high-definition front-end camera, touchpad, and microphone as well as a series of sensors (eg, accelerometer, gyroscope, Global Positioning System) [20]. Smart glasses are either connected to a mobile device (eg, mobile phone, tablet) or Wi-Fi network that enables access to the internet or a company’s information system. Although the use of a head-mounted display was evaluated by anesthesiologists in ORs to display vital signs more than 20 years ago [21], smart glasses eventually drew the attention of surgeons again in 2013 when Google released Google Glass. Due to the position of the camera next to the surgeon’s eye, it captures what the surgeon sees [22,23]. Within weeks of its distribution, Google Glass was being evaluated by surgeons in live-stream surgeries and was used to obtain advice from experts several thousand miles away. This device not only presents opportunities for medical students to visualize surgeries comfortably [16] but also for supervisors to evaluate junior surgeons [24]. Built on two recent literature reviews on the use of Google Glass in medicine [18,25], Table 1 summarizes and classifies a list of studies that took place in nonsimulated operative surgical settings.

Since 2013, many studies have investigated and demonstrated the ability of smart glasses to support surgeons in enabling remote diagnosis and assistance, documenting cases via photos or videos, and accessing patient information such as x-rays and vital signs, among other uses. However, while smart glasses are often cited in medical articles and scientific studies as potential candidates to address existing shortcomings with traditional checklist executions (ie, mostly the completion rate) [38,39], to our knowledge no research has empirically evaluated the use of smart glasses to execute checklists in a live OR setting.

**Table 1:** Existing research on the uses of smart glasses in health care.

Reference	Study design	Purpose of smart glasses use	Remote diagnosis and assistance	Video documentation	Photo documentation	Access patient information	(Self)-guidance
[26]	Pilot	Evaluate capacity of smart glasses to enhance communication, document cases, and access patient information	X		X	X	
[27]	Feasibility	Evaluate capacity of smart glasses to enhance communication and document cases	X	X	X		
[28]	Pilot	Use of head-mounted display on smart glasses to display vital sign parameters				X	
[29]	Pilot	List of smart glasses opportunities (eg, access to online medical encyclopedia, patient information, documentation, remote assistance)	X	X	X		
[30]	Case	Document cases and analyze pictures			X		
[31]	Pilot	Record first-person point-of-view video and photos and use as search engine		X	X	X	
[32]	Pilot	Live-stream video during surgery and facilitate remote telementoring between 2 surgeons, allowing real-time guidance of the operating surgeon	X				X
[33]	Feasibility	Assess the safety of using Google Glass by assessing the video quality of a telementoring session		X			
[34]	Pilot	Facilitate real-time observation and proctoring by mentoring surgeon experts in remote locations around the world	X				
[35]	Pilot	Enhance neuronavigation by projecting images directly on the Google Glass screen instead of traditional screens				X	X^a^
[36]	Pilot	Evaluate the use of Google Glass to document airway assessment and tracheal intubation			X		
[37]	Randomized controlled	Evaluate whether Google Glass can be used to perform an ultrasound-guided procedure					X

^a^Self-guidance was the secondary and not primary goal in surgery.

## Methods

### Context

We prospectively evaluated and compared 80 preoperative time-out events with SSCs at a major academic medical center between June 2016 and February 2017. Participants were assigned to either a conventional checklist approach (poster, memory, or both) or a smart glasses app (Google Glass). All surgical cases investigated were elective and gastrointestinal in nature. The hospital implemented the WHO SSC in 2009. This time-out checklist counts 13 items that must be verified by the surgeon responsible for the case; 5 additional items are used only when blood transfusion is part of the surgical procedure (which did not take place in this study). The checklist was customized according to the needs of the hospital and the different surgical departments. To ensure appropriate execution of the checklists, hospital checklist reporters attend random checklist executions (the checklist execution of a surgeon is monitored 1 to 4 times a month) and report their observations to the hospital’s administrators. To support surgeons in their checklist executions, the hospital has equipped its ORs with wall posters of the customized WHO checklist. Alternatively, surgeons can use a paper-based version of the checklist with the help of a circulating nurse.

### Checklist App for Google Glass

**Figure 1:**
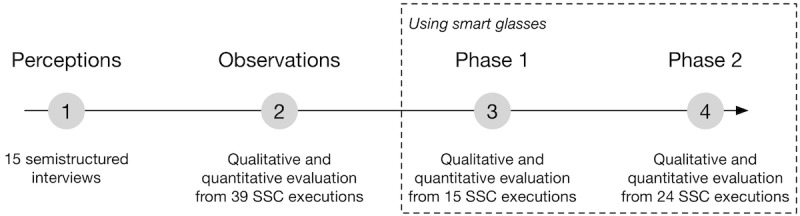
Data collection throughout the research process. SSC: surgical safety checklist.

To answer our research questions, we iteratively designed a smart glasses app following the action design research methodology [40], which promotes the involvement of end users in the design of the solution to ensure its efficiency and usability. As described below and shown in Figure 1, end users were involved from the needs definition to the evaluation of the smart glasses checklist app.

Our checklist app contained two screens: one to select the checklist and one to display the items of the checklist selected. In addition, we developed a checklist engine that allows for creating and maintaining individualized checklists. While checklist apps for smart glasses exist on the market, none of them provides the flexibility required for this study.

The app ran on smart glasses developed by Google, chosen for their popularity among surgeons and their versatile characteristics, such as a small screen that does not obstruct the surgeon’s field of view and, thus, does not disrupt communication among the OR staff. Additionally, they are very light and can work offline without any network connection.

### Approach

The research began with interviews of 15 surgeons to gauge their checklist execution experience and understand their challenges with traditional mediums. Data from the interviews also allowed us to compare their outcomes with the existing literature [9,12]. We then observed 41 conventional checklist executions in the OR, focusing on the way surgeons executed the checklists, completion rate, level of interaction with the OR staff, and duration. Leveraging the interviews and observation outcomes, we iteratively designed and refined a smart glasses app. When a new functionality of the app was ready, two surgeons were asked to evaluate it in a simulation room and make suggestions for refinements if the usability was too low (eg, difficult to read the items or navigate). Once the app met the requirements of the surgeons, it was evaluated in the ORs in two phases (Figure 1).

In phase 1, we evaluated the capacity of smart glasses to assist in executing the checklists, which involved only small wording adaptations in comparison with the official checklist (ie, poster). For phase 2, we collaborated with surgeons to customize surgery-specific checklists, following the recommendations by Weiser et al [41], in order to evaluate the benefits of having all the checklist items relevant for a set of surgeries. While it was clear which checklist items would be removed, it was not clear which new contextualized checklist items were appropriate for each checklist. Thus, it required several surgical cases to determine the relevant level of abstraction before including new checklist items. Surgeons also added items to ensure the readiness of specific equipment that is more likely not to be ready. Prior to the implementation of the smart glasses checklists, a 5-minute training was provided (only one surgeon had previous experience with Google Glass).

To evaluate the usability of smart glasses, we opted for a quantitative and qualitative research design. Using semistructured interviews, we were able to gather details about the ease of reading, following, and navigating through the checklist using smart glasses and modify the app based on surgeon feedback. We used descriptive statistics methods to evaluate the efficiency of the app.

### Data Collection

Data were collected at four distinct points throughout the study as shown in Figure 1. First, data from semistructured interviews were used to understand surgeon perceptions of checklist roles, benefits, drawbacks, and requirements. In total, 9 surgeons and 6 residents (at least postgraduate year 3) took part in the interviews. Second, during the observation phase, the authors attended and documented the execution of 41 conventional time-out checklists in ORs performed by 3 surgeons and 3 residents who also took part in the interviews. The documentation included the sequence and items verified by the surgeons, medium used for the checklist execution, level of interaction with OR staff (ie, low, medium, high), and duration. Third, in phase 1, the smart glasses app was used by 2 surgeons and 2 residents in the OR for a total of 15 surgical cases. After each case, the authors conducted a semistructured interview with the surgeon that focused on the ease of reading, following, and validating time-out items on the smart glasses, as well as the usefulness of the glasses to document the checklist executions and ensure their completeness. Each question was rated on a 4-point Likert scale and included a free-text field used to document additional comments. Last, in phase 2, surgeons used contextualized verifications by means of the smart glasses app in the OR for 24 surgical cases. The same evaluation process was used as in phase 1.

## Results

### Interview Outcomes (Perceptions) and Observation Results

The 15 interviewed surgeons agreed on the importance of time-out checklists to unite the OR staff and establish a common ground regarding the surgical procedure. However, as highlighted by existing studies, the surgeons found the current time-out mediums (ie, poster and paper) to be too generic, thus limiting their benefits. Similarly, the majority of interviewees found the mediums used to support the time-out execution difficult to read and follow. Therefore, surgeons often created their own and amended checklists that they could execute from memory. Alternatively, some surgeons started the time-out execution by memory and then used the poster to ensure they did not forget any verification.

### Usability of Smart Glasses to Execute Surgical Safety Checklists (Phase 1)

To answer the primary research question of whether smart glasses are a viable technology to execute time-out checklists, we analyzed the usability of smart glasses. We focused on the ease of reading, following, and navigating through a time-out checklist.

#### Ease of Reading Surgical Safety Checklist Content

**Figure 2:**
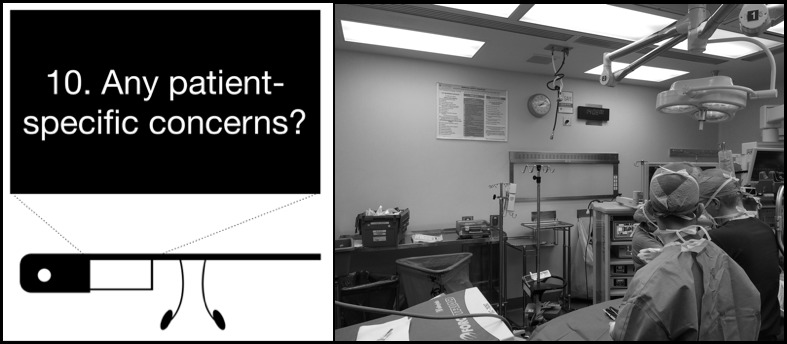
Checklist items displayed in the smart glasses app versus wall poster.

During the interviews, the majority of surgeons mentioned that the checklist wall poster is difficult to read and follow. With smart glasses, the checklist items appear in front of the surgeon’s eye. Although the Google Glass screen appears small, it renders a picture equivalent to a 25-inch high-definition television sitting 2.4 meters (8 feet) away. To enhance readability, only one checklist item is displayed in white on a black background, as shown in Figure 2. Participants strongly agreed that items on smart glasses are easy to read.

#### Ease of Following and Navigating Through the Surgical Safety Checklist

We observed on multiple occasions that surgeons lost their place during their time-out execution and had to cease the execution in order to find the next item on the poster. This issue was even more severe when surgeons commenced the checklist by memory and then forget their place. With the smart glasses, time-out items are displayed sequentially, requiring surgeons to go through all of the verifications. To mitigate this inflexibility, we offered surgeons the ability to create customized checklists by adding, removing, or editing any steps from those included in the basic checklist.

With regard to navigating within the checklist, voice commands were used in phase 1. The word “next” would trigger the next checklist item while “back” would return to the preceding item. The evaluation revealed 12 false negative events (ie, saying “next” with no change occurring). Tests revealed that the quality and sensitivity of the microphone were responsible for the low voice command recognition rate. In response, the interaction mode was changed to head gestures during the same phase 1 surgeries. The head gestures were the following: nodding up to down displayed the next checklist item and nodding down to up displayed the previous item. Finally, we decoupled the checklist items that originally contained multiple verifications. This was notably the case in the verification of the patient’s identity, position, and procedure. During the observation phase, surgeons forgot to verify at least one of the three items on 13 occasions. Surgeons either strongly agreed or agreed that checklists on smart glasses are easy to follow and navigate.

### Benefits of Smart Glasses to Execute Time-Out Checklists (Phase 2)

To investigate the benefits of smart glasses, we looked into their effect on the completion rate of time-outs and their ability to document checklist executions and contextualize the checklist contents.

#### Checklist Completion Rate

Existing studies largely demonstrate that incomplete checklist executions represent a critical problem that leads to complications in the OR [10,13]. As shown in Table 2, memory is the most common of all checklist execution means; however, it provides the lowest average completion rate (72%) and greatest average standard deviation (16%) across the execution (minimum completed items = 6; maximum = 13). It is interesting to note that surgeons executing time-outs by memory often missed different critical verifications despite the type of surgery being the same. When asked, no checklist executors realized they had forgotten items. By strictly following the poster, surgeons reached an average completion rate of 83% with the lowest average standard deviation (8%). However, critical verifications such as the identity and position of the patient and the use of deep vein thrombosis prophylaxis were sometimes forgotten. When surgeons relied on their memory and the poster, we observed they had significant difficulties in identifying the items they had already verified and those that remained. For this reason, they only performed slightly better than with memory, with an average completion rate of 77% and an average standard deviation of 10% (minimum completed items = 8; maximum = 13).

When surgeons used smart glasses in phase 1, the average completion rate increased to 98% with an average standard deviation of 3% (minimum completed items = 13; maximum = 14). Given that verifications appeared sequentially, the app forced surgeons to go through all the verifications. Surgeons did not complain about this when asked in the following interviews. We noticed that surgeons introduced themselves with their names but not their roles on three occasions in phase 1 (the only checklist item not performed at 100%). This behavior was also observed in the other mediums. When asked, surgeons said that they know the team and they do not want to repeat useless information.

#### Usefulness of Smart Glasses to Document Time-Out Executions

In our observation phase, we realized that time-out executions are not systematically documented. In our smart glasses app, we automatically document the time and checklist item each time the surgeon validates an item. Therefore, the time-out execution can theoretically be paired with the patient’s medical record. Given that this technique does not account for additional items that could be verified during the checklist execution, the entire time-out execution is recorded via the smart glasses microphone and stored in the device. All the participants strongly agreed on the usefulness of documenting the SSC executions.

**Table 2:** Checklist completion rates by recall medium.

Checklist item	Recall medium			
	Memory (n=20)	Poster (n=12)	Memory + poster (n=8)	Smart glasses (phase 1; n=15)
Correct patient	20 (100)	11 (83)	8 (100)	15 (100)
Correct procedure	17 (85)	11 (92)	8 (100)	15 (100)
Correct position	17 (85)	12 (100)	4 (50)	15 (100)
Correct operative site/side	1 (5)	6 (50)	0 (0)	15 (100)
Consent completed, accurate, and signed	16 (80)	12 (100)	8 (100)	15 (100)
Surgical site marked by surgeon and visible after preparation/after drape	3 (15)	9 (75)	5 (62)	15 (100)
Confirmation of allergies	19 (100)	12 (100)	8 (100)	15 (100)
Images/implants available	12 (60)	9 (75)	3 (38)	15 (100)
Prophylactic antibiotic given	20 (100)	12 (100)	8 (100)	15 (100)
DVT^a^ prophylaxis	18 (90)	10 (83)	8 (100)	15 (100)
Procedure duration	16 (80)	11 (92)	6 (75)	15 (100)
Any patient-specific concerns, are we all in agreement?	15 (75)	12 (100)	6 (75)	15 (100)
Is EBL^a^>500 cc or is there possibility of major blood loss?	18 (80)	10 (83)	8 (100)	15 (100)
Introduction by roles	11 (55)	1 (8)	6 (75)	11 (73)
Average	14.4 (72)	10 (83)	6.2 (77)	14.7 (98)
Standard deviation	3.2 (16)	1 (8)	0.8 (10)	0.45 (3)

^a^DVT: deep vein thrombosis.

^a^EBL: estimated blood loss.

#### Implementation of Contextualized Checklists to Increase Checklist Benefits

One of the main issues when using static mediums such as posters and papers lies in the inability of the checklist content to be adapted. While each surgical procedure type requires the verification of specific items, such as surgical phases and equipment, our interviews revealed the paper-style checklists could only provide very generic items. To evaluate the benefits of customized checklists, we developed five checklists that represented the most frequent surgery types we investigated in this research: laparoscopic sleeve gastrectomy, laparoscopic gastric bypass, esophagogastroduodenoscopy, per-oral endoscopic myotomy, and laparoscopic cholecystectomy. In addition, we numbered each of the verifications to indicate the progression in the checklist and moved the “introduction by role” to the first item, as suggested by WHO.

**Figure 3:**
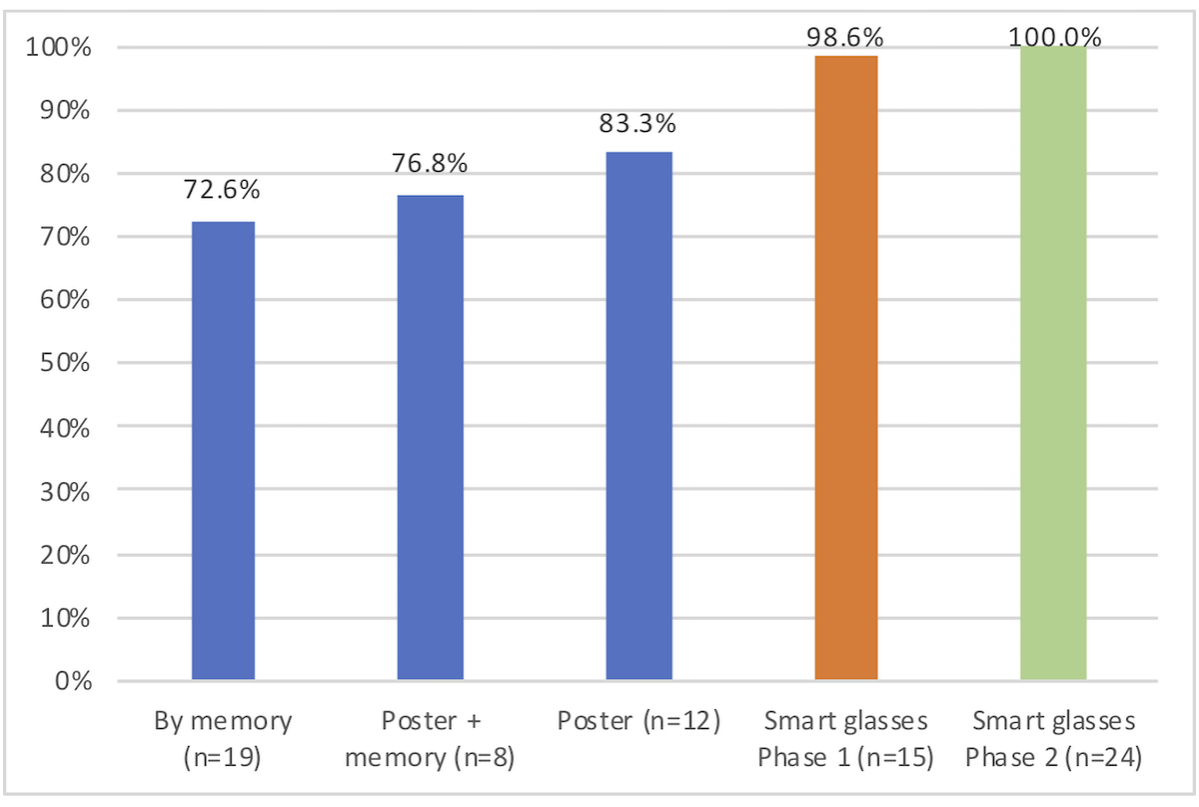
Completion rates across all mediums.

**Table:** 

	Memory	Memory + poster	Poster	Smart glasses (phase 1)	Smart glasses (phase 2)
Average duration (seconds)	54.8	58.5	60.8	85.7	47.7
Standard deviation (seconds)	10.9	14.6	15.6	28.5	13.2

After 24 checklist executions with the smart glasses in phase 2, the completion rate was 100% as shown in Figure 3. Contextualized checklists also had an impact on the checklist duration given that only relevant checklist items were verified. Our evaluation revealed that contextualized checklists via smart glasses required less time compared with using the generic checklist, as shown in Table 3. It is not surprising to observe that executing the checklist for the first time with Google Glass required some adaptation time. However, making lists more relevant as they were contextualized resulted in decreased checklist duration and preserved high completion rates of all relevant items.

From the interviews, all participants strongly agreed that the use of contextualized checklists brings more efficiency to checklist executions.

## Discussion

### Principal Findings

In this research, we evaluated the use of smart glasses to improve the completion rate and, hence, the benefits of time-out checklist executions. In interviews, surgeons stressed the importance of differentiating between voluntary versus involuntary checklist noncompletion. The first is legitimate and happens when the checklist and the context are not aligned (eg, patient-specific items to verify). The latter occurs when surgeons unintentionally exclude items. Our evaluation confirms that smart glasses have the capability to address both types of noncompletion. They not only improve the completion rate but also increase the checklist’s relevance and benefits. We observed that the checklist content differed by up to 40% across the selected checklists of this study. The implementation of smart glasses to contextualize SSCs allows deliberate list customization for specific surgeons and procedures, preventing the need for voluntary checklist noncompletion. At the same time, surgeons do not experience the need to improvise any additional checklist steps on the go as required when reading from a standardized list that does not adapt to real procedural circumstances. Furthermore, our results also show that the use of smart glasses was particularly relevant when no poster was available. Certainly, smart glasses had some hindrances; for example, we observed that they tended to isolate surgeons from the rest of the OR staff and that surgeons would move and interact less when using smart glasses. It is unclear whether this was a result of ergonomics, visual constraints, or just the awareness of wearing a novel head-mounted display.

Questions pertaining to implementation costs, maintenance, and use of such devices remain open. To the best of our knowledge, no hospitals have been using smart glasses at a large scale on a regular basis. Potential problems related to cleaning the device, overall reliability, and fragility are responsibilities that hospitals will soon have to accept. Beyond the US $1500 cost of each Google Glass headset, simple implementation decisions can also influence costs (eg, whether to adopt wired or wireless update and information transfer). While most ORs are equipped with wireless connectivity, for safety reasons, data must be encrypted, which increases the complexity. Shall the hospital equip each OR or each surgeon? If the former, the OR staff will be responsible for cleaning, updating, and ensuring that the device functions when the surgeon requires it. If the latter, the surgeon would be responsible for the device.

### Limitations

This research had some limitations. There was no randomization in this study. While randomization could have been achieved fairly simply, there was limited availability of smart glasses and of some key members of the research team. Perhaps in the near future, these described limitations could be overcome with universal availability of this technological platform.

### Conclusion

Our results show that smart glasses have the capacity to yield checklist completion rates of 100% by providing better usability than traditional mediums. Another benefit of using smart glasses lies in the use of contextualized checklist items. These allow surgeons to focus on context-relevant verifications while irrelevant checklist items are removed. In addition, smart glasses can be used to automatically document and transfer checklist executions to the patient’s medical record.

Beyond smart glasses, this research also demonstrates the inefficiency of merging multiple verifications into one checklist item. Our observations show that most of the forgotten verifications are those that are merged with others.

This research is a first step toward clinically evaluating the efficiency of smart glasses in a long-term study. We encourage researchers and clinicians to further evaluate the use of smart glasses to execute checklists in surgery and other interventional procedures.

## Abbreviations

OR: operating room
SSC: surgical safety checklist
WHO: World Health Organization
